# Factors That Drive Dentists towards or Away from Dental Caries Preventive Measures: Systematic Review and Metasummary

**DOI:** 10.1371/journal.pone.0107831

**Published:** 2014-10-08

**Authors:** Uhana Seifert Guimarães Suga, Raquel Sano Suga Terada, Adriana Lemos Mori Ubaldini, Mitsue Fujimaki, Renata Corrêa Pascotto, Adelia Portero Batilana, Ricardo Pietrobon, João Ricardo N. Vissoci, Clarissa G. Rodrigues

**Affiliations:** 1 Department of Dentistry, State University of Maringá, Maringá, Brazil; 2 Department of Surgery, Duke University Medical Center, Durham, North Carolina, United States of America; 3 Department of Medicine, Faculdade Ingá, Maringá, Brazil; 4 Instituto de Cardiologia do Rio Grande do Sul - Fundação Universitária de Cardiologia (IC-FUC), Porto Alegre, Brazil; LSU Health Sciences Center School of Dentistry, United States of America

## Abstract

**Background:**

Dental caries is a serious public health concern. The high cost of dental treatment can be avoided by effective preventive measures, which are dependent on dentists’ adherence. This study aimed to evaluate the factors that drive dentists towards or away from dental caries preventive measures.

**Methods and Findings:**

This systematic review was registered in PROSPERO (CRD42012002235). Several databases as well as the reference lists and citations of the included publications were searched according to PRISMA guidelines, yielding 18,276 titles and abstracts, which were assessed to determine study eligibility. Seven qualitative studies and 41 surveys (36,501 participants) remained after data extraction and interpretation. A total of 43 findings were abstracted from the reports and were grouped together into 6 categories that were judged to be topically similar: education and training, personal beliefs, work conditions, remuneration, gender, place of residence and patients. The main findings for adherence based on their calculated frequency effect sizes (ES) were teamwork (21%) and post-graduation (12%), while for non-adherence were biologicism (27%), and remuneration for preventive procedures (25%). Intensity ES were also calculated and demonstrated low prevalence of the findings. Quality assessment of the studies demonstrated that the methodological quality, particularly of surveys, varied widely among studies.

**Conclusions:**

Despite the questionable quality of the included reports, the evidence that emerged seems to indicate that further education and training coupled with a fairer pay scheme would be a reasonable approach to change the balance in favor of the provision of dental caries preventive measures by dentists. The results of this review could be of value in the planning and decision making processes aimed at encouraging changes in professional dental practice that could result in the improvement of the oral health care provided to the population in general.

## Introduction

Dental caries is considered a serious public health problem with significant impact on the quality of life that causes pain and suffering, leads to the loss of school and working hours and affects social relationships [1]. Dental caries is still one of the most prevalent diseases of the oral cavity, afflicting 60 to 90% of school-age children and the vast majority of adults in industrialized countries [1], [2]. The distribution of oral diseases varies among different parts of the world and within the same country or region [3], and according to the availability and accessibility of oral health services [1]. Dental caries is a most prevalent oral disease in several Asian and Latin-American countries, while it appears to be less common and less severe in most African countries [3]. Risk factors for oral diseases include an unhealthy diet, tobacco use, harmful alcohol use and poor oral hygiene, as well as social determinants [1]. In all countries, the oral disease burden is significantly higher among poor and disadvantaged population groups [4].

The treatment of dental caries requires restorative procedures that represent a significant cost in many high-income countries, where oral health can account for 5 to 10% of all public health expenditure [4]. For the majority of low-income nations, the cost of treating caries with the traditional method of restorative dentistry is beyond their financial capabilities, as most of these countries can not finance an essential package of health care services for their children [5]. The high cost of dental treatments can be avoided and caries prevalence can be more effectively tackled by effective prevention and health promotion measures. However, although information on the various etiological factors involved in the development of caries and strategies for its prevention have become widely available, much of the population in many parts of the world is still affected by the disease [6], and the global incidence of dental caries in school-age children remains high [4].

It has already been shown that dentists have poorly contributed to the reduction in the prevalence of dental caries [7]. However, dentists have the potential to influence what their patients know and do regarding dental caries prevention [8], and are often necessary especially in individual prevention. Knowing the reasons that drive dentists away from performing prevention and those that facilitate its adoption can bring an important contribution towards the implementation of dental caries preventive programs.

Thus, the aim of this systematic review was to analyze studies that have investigated the factors that drive dentists towards or away from dental caries preventive measures and conduct a metasummary of the results found.

## Materials and Methods

### Protocol and registration

This systematic review was carried out in accordance with the Preferred Reporting Items for Systematic Review and Meta-Analyses (PRISMA) Statement [9], and was registered in the International Prospective Register of Systematic Reviews (PROSPERO) under the registration number CRD42012002235.

### Eligibility criteria

The inclusion criteria were as follows: 1) publications methodologically designed as a “qualitative study” or “survey”, qualitative studies classified as those whose findings were abstracted from unstructured data, i.e., individual or group interviews, while surveys were those studies whose findings were compiled from structured questionnaires; and 2) publications reporting factors that drove dentists (public and private) towards or away from incorporating dental caries preventive measures in their practice. The following exclusion criteria were applied: 1) publications in which the research subjects were dental technicians, doctors, nurses or dental students, and 2) publications presenting factors related to preventive measures such as the use of sealants, mouthrinses, or water fluoridation investigated in isolation.

### Literature search

The following electronic databases were used for the selection of the primary studies: PubMed, EMBASE, PsycoInfo, Scielo, Scopus, Web of Science, BBO, Lilacs and York. To ensure the widest possible search, no language filters were applied [10]. The reference lists of the retrieved studies were searched for additional publications, and the citations were also analyzed using Google Scholar. The authors of included studies were contacted by email for the identification of additional studies.

### Search strategy

The following terms were used in the search strategy: “dentist”, “dentists”, “general dental practitioner”, “general dental practitioners”, “dental caries”, “prevention”, “oral health”. MeSH terms were used along with the listed entry terms to construct a highly sensitive search strategy. Terms related to the study type were not used because the term “qualitative research” was only introduced in EMBASE in 1988 and as a MeSH term in PubMed in 2003. The complete search strategy used for the PubMed database is shown in Appendix S1.

### Study selection

Two reviewers (USGS and ALMU) independently read all retrieved titles, abstracts, and full-text articles. If one assessor regarded a publication as having met the inclusion criteria, the full text was obtained. Abstracts considered as potentially eligible, as well as those that did not supply enough information, were reserved for the assessment of the full-text article. Any differences concerning eligibility after the full text was evaluated were resolved through consensus, and when differences still persisted, a third reviewer (RSST) was consulted before a final decision was reached.

### Quality assessment

The quality of the selected studies was assessed by classifying each study according to items adapted from Bennett et al., (2010) [11] for surveys, and the Joanna Briggs Institute Qualitative Assessment and Review [12] for qualitative studies.

The quality assessment of included surveys considered the inclusion of the following items: i) research question justification; ii) explicit research question; iii) clear objectives; iv) description of the methods used to analyze data; v) method used to administer the research instrument (questionnaire); vi) place and date of the study; vii) method described well enough to be replicated; viii) reliability of evidence; ix) validity of evidence; x) method used to verify data entry; xi) use of codification; xii) sample size calculation; xiii) method for selecting the sample; xiv) description of the study population; xv) description of the research instrument; xvi) description of the research instrument development; xvii) instrument pre-test; xviii) instrument reliability and validity; xix) scoring method; xx) informed consent obtained; xxi) ethics approval; and xxii) evidence of ethical treatment of research participants; and xxiii) sample representativeness.

The items analyzed in the qualitative studies were: i) correspondence between the methodology and the indicated philosophical perspective (theory); ii) correspondence between the methodology and the research question or objective; iii) correspondence between the methodology and the methods used for data collection; iv) correspondence between the methodology and data presentation and analysis; and v) correspondence between the methodology and interpretation of the results. Other considerations included statements that: vi) placed the researcher culturally or theoretically; vii) indicated researchers’ influence on the study or vice-versa; viii) demonstrated the representation of participants and their voices; ix) showed the investigation was ethically performed according to current criteria or, in more recent studies, the evidence of ethical approval by recognized institutions; and x) indicated that conclusions were drawn from research reports or from data analysis or interpretation.

The items above were verified and classified as definitely present (yes), partially or unclearly present (not clear), or definitely not present (no). Studies that presented a prevalence of “yes” answers (>50%) in the quality assessment were deemed to have a low risk of bias, studies that did not clearly present many of the items assessed were classified to have a moderate risk of bias, while studies that presented a prevalence of “no” answers (>50%) were considered to have a high risk of bias.

### Data extraction

Two reviewers (USGS and ALMU) independently conducted data extraction. General information such as authors, year of publication and first author geographic region were collected from each study. Additionally, the following specific characteristics were also collected: objective, type of study, place where the research was carried out, interventions, number of participants in the sample, inclusion and exclusion criteria, participant characteristics, data collection, data analysis, main results, and authors’ conclusions.

### Data analysis

Qualitative metasummary is a quantitatively oriented aggregation of qualitative findings originally developed to accommodate the different characteristics of qualitative studies and surveys [13]. Qualitative metasummary includes the extraction, grouping, and formatting of findings and the calculation of frequency and intensity effect sizes (ES), which permits to produce mixed research syntheses and to conduct a posteriori analyses of the relationship between reports and findings [13].

After the extraction of results from the included studies and the grouping of relevant findings, categories (concise but comprehensive representations) concerning the factors that drive dentists towards or away from carrying out dental caries preventive measures were developed. The categories concerned not only dentists, but also their views of how the factors studied affected their patients. Qualitative data analysis software (ATLAS.ti 7) was used to codify the themes that emerged from the analysis.

To assess the relative magnitude of the extracted results, frequency ES was calculated by taking the number of studies containing a particular finding (minus the studies derived from a common parent study and representing a duplication of the finding) and dividing this number by the total number of included studies (minus the reports derived from a common parent study and representing a duplication of the finding), and expressed as a percentage.

After that, to ascertain which findings reports contributed to the final set of abstracted themes, intensity ES of each report was also calculated. This information is useful for various a posteriori analyses: for example, to determine whether any findings were derived from largely “weaker” studies, which reports contributed most of the findings with the largest frequency effect sizes across reports, and which reports contained findings no other reports contained. Intensity ES calculation was performed by: i) dividing the number of findings contained in the study by the total number of findings across all studies; and ii) by dividing the number of findings with effect sizes >25% contained in that study by the number of findings with effect sizes >25% across all studies.

## Results

### Study selection

**Figure 1:**
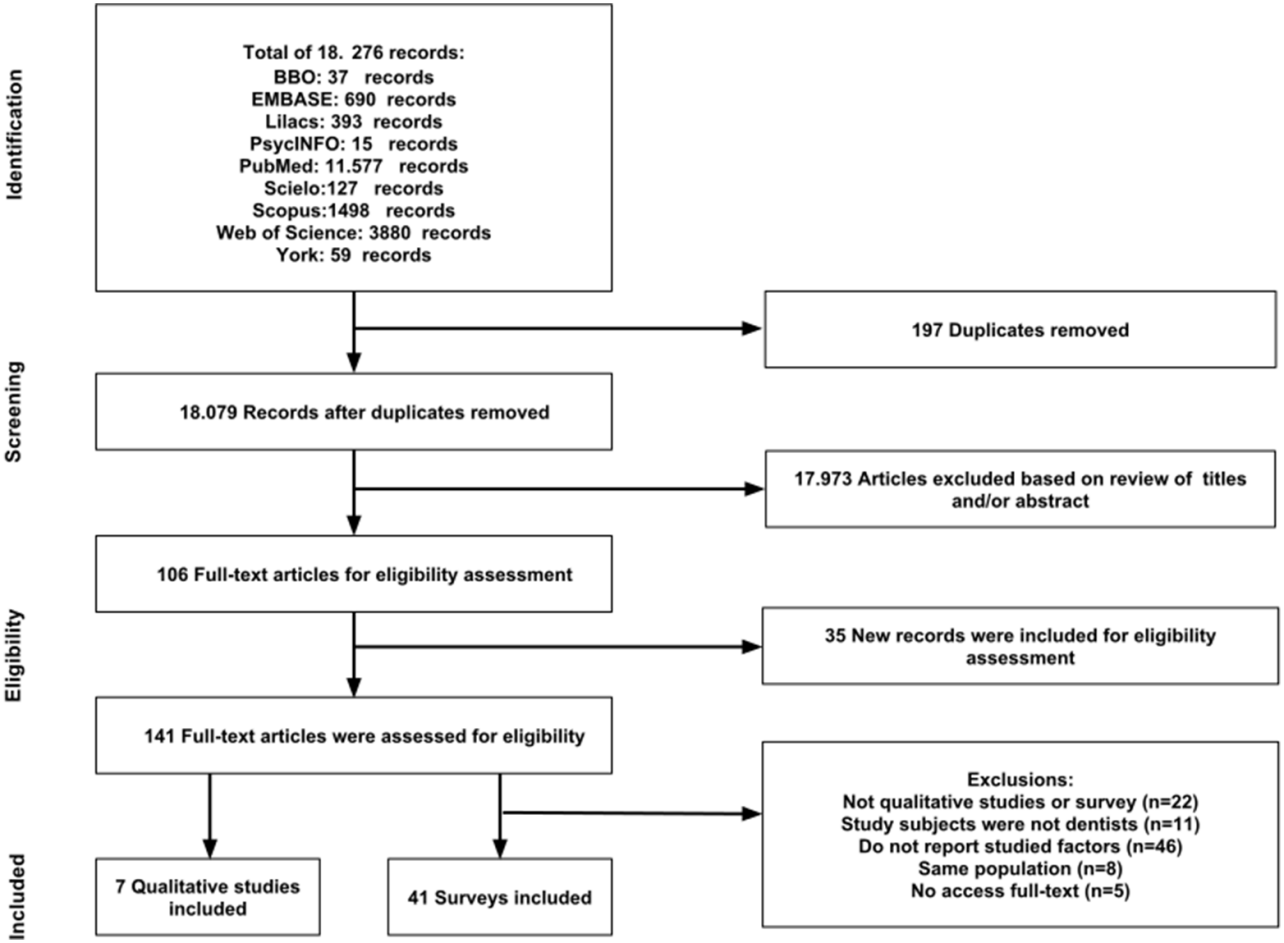
Flowchart showing the number of publications identified, retrieved, extracted, and included in the final analysis.

The search of electronic databases yielded 18,276 references. After removing duplicates and assessing titles and abstracts, 106 publications were considered potentially eligible. Full texts were retrieved and analyzed for eligibility. After analysis of the reference lists and Google Scholar citation, 35 additional publications were selected and their full text retrieved and analyzed. Ninety-two publications were excluded for the following reasons: 1) the design of 22 publications did not meet the criteria of a “qualitative study” or “survey”; 2) the research subjects in 11 publications were not dentists; 3) 46 publications did not report the factors that drive dentists towards or away from dental caries preventive measures; 4) the full text of five publications could not be retrieved; and 5) eight publications presented the same sample population. A total of 48 publications were selected comprising seven qualitative studies [14]–[20] and 41 surveys [8], [21]–[60]. The electronic contact with authors of included publications did not result in any additional studies. Figure 1 summarizes the process of literature identification and selection.

### Study characteristics

Information on the included studies (sampling, intervention, objectives, outcome and risk of bias) is presented in Table 1 (qualitative studies) and Table 2 (surveys). The total number of participants in the seven qualitative studies included in this review was 390. The studies presenting the largest number of participants (311) were Threlfall et al. 2007 [19] and Threlfall et al. [20]. Other included qualitative studies presented populations that varied between 2 and 28 participants. The total number of individuals participating in the selected surveys was 36,111. Surveys, contrary to qualitative surveys, presented a much wider range of participants, varying from as few as 15 [42] to as many as 4,850 [22].

**Table 1:** Publication characteristics of the qualitative studies included in the analysis.

Study	Sampling	Intervention	Objectives	Outcome	Risk ofbias
Cashmore AW et al, 2011(Australia) [14]	8 program dental staff and 2 co-ordinating staff	Focus group interviews and semi structured interviews	To explore the attitudes and beliefs of dental staff about the factors that helped or hindered the establishment and implementation of a hospital-based parent counseling program to manage existing, and prevent new, carious lesions in children	The participants identified a number of factors that they feltinfluenced the establishment and implementation of the program, including the dental team’s support of the initiative, the advantages of building on existing clinic infrastructure and procedures, the utility of harnessing dental assistants as a resource for oral health promotion, and the confidence of dental professionals to provide parent counseling.	Low
Gussy MG et al, 2006(Australia) [15]	22 dental professionals working in the four local government areas.	Qualitative focus group discussionsand semi-structuredinterviews	To explore the oral health beliefs and practices of primary health care professionals which may act as barriers to the development of a model of shared care for the oral health of pre-school children.	Dental professionals did not believe that they had a primary role in the oral health of pre-school aged children but those others particularly maternal and child health nurses did. However other health care professionals were not confidentin assuming this role.	Low
Humphreys REet al, 2010(Wales) [16]	19 First yearfoundation dentitsin South wales	Focus groupdiscussions	To explore the perceptions of first year foundation dentists (FD1s) regarding oral health education (OHE)and its role in general dental practice.	OHE is often compartmentalized and a simplistic approach to its delivery is taken. Against a backdrop of commissioning to improve health this has implications in developing organizational processes within general dental practice and training in order to achieve this.	Low
Nettleton S et al, 1989(England) [17]	28 Communitydentists	–––	To describe the perceived problems and difficulties of 28 community dentists when carrying out dental health education	Before enthusiastically endorsing dental health education in the dental surgery it is necessary to clarify what the people involved understand by it, and the extent to which they are willing and able to adopt new practices	Moderate
Sbaraini A et al, 2012(Australia) [18]	8 Generaldental practicesin Australia	Participants were interviewed for approximately one hour in locations convenient to them.	What factors influence a general dental practitioner to offer preventive care to patients?	The key conditions needed for practices to reorient to preventive care included the presence of a committed leader with a prevention-supportive peer network, and the reorientation of space, routines and fee schedules to support preventive practice.	Low
Threlfall AG et al, 2007(England) [19]	93* general dental practitioners practicing within the general dental service in North West of England	Semi-structuredinterviews	To increase understanding about how and to whom general dental practitioners provide preventive advice to reduce caries in young children.	Children with caries were more likely to be questioned about diet and oral hygiene and if dentists believed parents to be motivated they were more inclined to spend time providing advice. Most dentists seemed to believe that education was the key to preventing caries and gave preventive advice in the form of a short educative talk.There was little use of visual aids or material for parents to take home.	Low
Threlfall AG et al, 2007(England) [20]	93* (see above)	Semi-structuredinterviews	To increaseunderstanding about the content of preventive advice and care offered by general dental practitioners to young children	Preventive advice given to parents of young children is usually about sugar consumption and tooth brushing behavior but the emphasis and specific messages provided varies among general dental practitioners.Use of fluorides varied considerably, suggesting that some dentists either have reservations or are unclear about the appropriate use of fluorides.	Low

*Same population. Number of participants counted only once.

**Table 2:** Publication characteristics of the surveys included in the analysis.

Study	Sampling	Intervention	Objectives	Outcome	Risk ofbias
ADAHF, 1984,(USA) [21]	4000 Dentists identified in the American Dental Associatiońs master file as actively in private practice, including specialists	mailed surveyquestionnaire	To present the results of a preventive dentistry survey.	The current thinking of dentists regarding caries prevention, as reflected by attitudes toward and use of procedures, is not entirely consistent with the state-of-the-art or consensus positions of the academic and research communities. Although practitioners are aware of the primacy of community water fluoridation for caries prevention, they do not appear to fully appreciate the values of other fluoride modalities, including topical fluorides, dentifrices, and mouthrinses. They especially undervalue pit and fissure sealants.Conversely, practitionersemphasize oral hygiene and diet counseling procedures for caries prevention despite scientific reports questioning their worth in this regard.	High
Ananaba N et al, 2010(USA) [22]	137 general and 45 pediatric dentists in Michigan and 2,112 pediatric dentists outside Michigan	questionnaire	To explore attitudes and behavior concerning IOHE among general and pediatric dentists in Michigan and pediatric dentists in the remaining 49 U.S. states.	General dentists had more negative attitudes towards IOHE than pediatric dentists in Michigan and other U.S. states.Only 41% of general dentists vs. 84% and 89% of pediatric dentists in Michigan and other states performed IOHE.While general dentists who performed IOHE had better attitudes towards IOHE than their non-IOHE-performing colleagues, they engaged less in prevention directed activities compared to pediatric dentists.	High
Anderson R et al, 2002(Wales) [23]	1160 identified dentalprofessionals workingin Wales	questionnaire	To provide a comprehensive profile of the current nature and scale of health promotion by dental professionals in Wales.	Acceptable and achievable goals of effective preventive practice should be informed by evidence of what practitioners currently do and currently believe as well as the evidence of what is shown to be effective.	High
Badan DEC et al, 2010(Brazil) [24] *	72 recently graduatedstudents from UFG Dental School from 2000 to 2002.	e-mailedquestionnaire	To know the perceptions and uses of the collective health knowledge in thedaily practice of the dentists in the period	Dentists showed doubts about collective health actions in spite of saying that they practiced them.They reported the lack ofcomplementary material and little valorization by the population of prevention activities. Collective health practices should be made clearer and deeper, and curriculum integration should take place during dental courses.	High
Brennan DSet al, 1996(Australia) [25]	202 private general practitioners who provided service rate data in both 1983 and 1988	questionnaire	To establish dentist practice styles and to assess the distribution of these styles of practice between 1983 and 1988	Net movement away from the “High restorative” “ and “ “Low Total Rates” “ clusters toward the “High Diagnostic and Preventive” “cluster was shown, but there was movement by practitioners away from and into all clusters.	low
Brennan DS et al, 1998(Australia) [26]	A random sample of Australian dentists in 1983–84, 1988–89, and 1993–94.	questionnaire	To identify trends in service provision over time.	Findings indicate changing patterns of practice over time, consistent with an increasing orientation towards prevention of disease and maintenance of a natural dentition.	High
Brennan DS, et al. 2001(Australia) [27]	345 from a random sample of Australian dentists	mailed questionnaire	To replicate practice belief scales in Australia and investigate associations withdentist and practice characteristics and services.	The findings confirm the factor structure of practice beliefs and demonstrate small to moderate associations with variation in service rates.	Low
Brennan DS et al, 2003(australia) [28]	489 random sample of dentists from each State/Territory in Australia in 1998–99	mailed questionnaire	To examine the provision of examinations, radiographs, prophylaxis and topical fluoride, and to assess whether these services varied by patient, visit and oral health characteristics.	Radiographs may often be used to confirm disease rather than in early detection, and prevention was mainly provided to asymptomatic patients in routine maintenance schedules. Many emergency patients and those with oral diseases presented missed the benefits of prevention.	High
Brennan DS & Spencer AJ, 2007(Australia) [29]	NA - A random sample of dentists in 1983, 1988, 1993,1998 and 2003	mailedquestionnaire	To investigate time trends in dental service provision by location.	While the overall content of dentist workloads has changed to include less emphasis on removal and replacement of teeth and more effort on diagnosis and prevention aimed at retention of natural dentitions, a gap by location remains, with dentist workloads outside of major city locations marked by higher rates of tooth extraction and lower rates of preventive services.	High
Calnan M et al, 2000(England) [30] *	1956 dentists with open General Dental Service contracts	questionnaire	To explain why some dentists have changed private/public mix, and why private practice appears to be increasingly attractive.	The movement toward selective NHS dentistry might be perceived as an appropriate solution by dentists, but evidence suggest that is not acceptable to the public in general or to the users of dental services	High
Chen M,1990 (USA) [31]	1000 dentists in general practice and pedodontics in Texas, USA, who were registered with the Texas Board of Dental Examiners as of 1984.	questionnaire	To present the results of a 1985 survey of 1000 Texas dentists regarding three major types of preventive measures–educational services, preventive procedures, and diagnostic services.	Among preventive procedures, most dentists removed plaque or calculus. Income, attendance of continuing education programs, and number of dental hygienists were strong, positive predictors of provision of all three types of preventive measures.Dentists who practiced in more populous areas, or had practiced for fewer years, more likely provided patients with educational services and preventive procedures.Dentists delivered more preventive procedures if they attended more professional dental meetings. Dentists who worked more hours were more likely to provide educational services and preventive procedures.	High
Chestnut IGet al, 2007(Wales) [32]	691 general dentalpractitioners in Wales	mailedquestionnaire	This study investigated the perceptions and attitudes to the new contract, in the three months immediately prior to its implementation.	This study has established baseline perceptions of reform in state-funded dental care in Wales. As the new contract evolves, it will be interesting to determine whether the largely negative perceptions of new ways of working expressed in this study are realised.	Low
Craft M et al, 1976(Australia) [33]	502 practitioners who worked in 6 comparable towns, 3 from the North and 3 from the South of England	questionnaire	Five areas of practice that were likely to be affected by differing attitudes to prevention were studied: (l) fluoridation; (2) employment of ancillaries; (3) professional life and self-image; (4) prevention in patients; (5) practitioners own immediate family.	The findings tended to show that in parallel with studies of regional distribution of treatment and services, more negative attitudes to prevention are found in areas with fewer services and poorer treatment patterns, and vice-versa	High
Fiset Let al, 1997(USA) [34]	532 Washington Stategeneral dentists	questionnaire	Dentists were surveyed about their use of four caries-control services among adult patients.	Leaders in the dental community and those with a wider network of professional colleagues were likely to adopt new services more quickly than other dentists.Earlier adopters also had more correct information about these services than later or no adopters.	Low
Freeman R et al, 2005(NorthernIreland) [35]	166 General dentalpractices located within the region of the EHSSB (Northern Ireland Eastern Health and Social Services Board)	questionnaire	To investigate the preventive orientation of general dental practices by examining their patient-active prevention activities, practice policies for prevention and employment strategies.	The findings suggest that the employment of a hygienist is central to the reorientation of primary dental care. TheGovernment must be encouraged to provide the financial means to allow primary care to shift from being disease-centered to health-focused.	Low
Ghasemi Het al, 2007(Iran) [36]	1033** dentistswho participated in two annual dental meetings in December 2004 and in July 2005, in Tehran, Iran	self-administeredquestionnaire	To assess Iraniandentists’ knowledgeof and attitudes towards preventive dental care.	Dentists’ knowledge of and attitudes towards prevention should be improved and updated to enable and encourage them to provide their patients with preventive care.	High
Ghasemi Het al, 2008(Iran) [37]	1033** (see above)	self-administeredquestionnaire	To study risk-basedpreventive practiceamong Iranian dentists.	To better meet each patient’s need, more emphasis on a risk-based approach in preventive dental care is called for in dental school curricula and continuing education. In this process, comprehensive guidelines for preventive practice would be helpful.	High
Ghasemi Het al, 2009(Iran) [38]	1033** (see above)	self-administeredquestionnaire	To examine the perceivedbarriers to preventivedental practiceamong Iranian dentists.	Dentists recognized a broad range of factors as barriers to the provision of preventive dental care, the strongest addressed to the patient-related barriers. The perceived barriers to the provision of preventive care should be investigated in greater detail and tackled to enhance oral health in Iran.	Low
Grembowski Det al, 1990(USA) [39]	200 general dentistsbased on a homogeneous,well-educated, upper-middle-classpopulation of patients	questionnaire	Factors influencingvariation in dentist service rates	Results indicate that practice characteristics, patients’ exposure to fluoridated water supplies, and the extent of no price competition in the market influence the services that patients receive.Therefore, attempts to address these issues will necessarily involve altering dentists’ decisions regarding practice organization and the delivery of care. However, because these factors account for less than 30 percent of the variation in the rates, the future impact of any single intervention may be limited.	Low
Holloway P Jet al 1994(England) [40] *	50 successful,general dentalpractitioners	questionnaire	To discover whatpreventive procedureson which patientsconsidered were the benefits of theirpractices and why.	All dentists thought that prevention on selected patients was of value to their practice.They said that prevention enhances the reputation of the practice, adds to the job satisfaction of the dentistand is part of modern dental philosophy. However, only when practised selectively would it be cost-beneficial. Dentists who employed hygienists had a significantly higher ‘mean preventive awareness score’ than those who did not.	High
Kallestål Cet al, 1999 [41]	Random samplesof dentists, dentalhygienists, anddental nurses workingwith children during1995 and 1996	questionnaire	To compare thecaries-preventivemethods used forchildren and adolescents inDenmark, Iceland,Norway, and Sweden.	Informational basis of decisions on preventive strategies varied between the different dental professionals in each country as well as between the countries, indicating that national professional cultures are being shaped differently. Despite the differences in choice of preventive methods, the dental health of children varies little across the frontiers.	High
Kay EJet al, 2003(England) [42]	15 Fifteen generaldental practicesconducting a simultaneous survey of attending patients and 15 practitioners fromthese practices.	Mailedquestionnaire	To measure thesubjective impactof oral health in a groupof patients attendinggeneral dental practices in the North West of England and to investigate the attributes of dentistsand practices in order to examine how such attributes might relate to patients’ subjective perceptions of oral health.	Fourteen percent of the differences in patients’ subjectively perceived oral health can be attributed to dentist attitudes and attributes. Further research regarding the influence of dentists personality and professional beliefs on patients well-being needs to be undertaken.	High
Malcheff Set al, 2009(USA) [43]	2157 membersof the AAPD	questionnaire	To: (1) determine pediatricdentists’ behaviors and attitudes concerning infant oral health examinations (IOHEs); and (2) explore how respondents who do or do not perform IOHEs differ in their behavior and attitudes concerning IOHEs.	The finding that only 53% of the respondents see 1-year-old children or younger shows that efforts need to continue to increase the percentage of dentists who offer IOHEs. Most respondents held rather positive attitudes toward IOHEs. They differed in the amount of time they schedule for these exams and the issues they address.	High
Milgrom P et al, 1988(USA) [44]	521 general practitioners in Washington State.	questionnaire	To investigate dentist attitudes and activities regarding oral self care.	Though most dentists say they counsel patients about oral self care, when specific practices are reported it was found that only a small percentage actually utilize an approach that would be considered effective.	Low
Moon H et al 1998(Korea) [8]	2,047 dentists, selected by a stratified random sampling allocated proportionately	a pretested, 27-item questionnaire	To determine thelevel of knowledge and opinions about caries etiology and prevention among Korean dentists and to describe related factors.	The majority of dentists do not know current information concerning etiology and prevention of dental caries, mechanisms of action of fluoride, and effectiveness of preventive procedures for children and adults. Efforts to enhance the level of knowledge and practices of Korean dentists about caries prevention should focus on strategies to educate older graduates and female dentists, especially those in private practice.	Low
Murtomaa H et al 1988(Finland) [45]	570 dentists registered on the Finnish DentalAssociation	questionnaire	To investigate deattitudes of practitioners towards health dental education and their opinion about its development anddifficulties related to it	Younger dentists were less in favor than their older colleagues of increasing the amount of health education. −9% considered that health education should be carried out only by auxiliaries. -Older dentists recognized moreoften than younger colleagues that the lack both material and individual resources were a problem in health education.	High
Nuca CI et al, 2011(Romania) [46]	348 dental practitioners registered to practice in Constanta, Braila, Galati, Tulcea, Buzauand Vrancea districts.	questionnaire	To evaluate the current working practices in preventive dentistry of dentists from six Romanian districts–the South-East Romanian Development Region.	The results of this study demonstrate the need to increase the awareness and skills of dentists from the South-East Development Region of Romania regarding the prevention of oral diseases, especially in terms of cross-infection control in dentistry, in order to meet European Union standards and to ensure health and safety at work in dentistry.	Low
Pine CM,et al 2004 [47]	2,333 dentists in 14 countries (Belgium, China, Czech Republic, Denmark, Germany, Ireland, Madagascar, Mexico, Singapore, South Africa, Tanzania, Thailand and USA) and 17 sites	questionnaire	To explore whether dentists’ beliefs and attitudes to providing preventive and restorative dentalcare for young children can form a barrier to the provision of care.	In most countries, dentists agreed that young children’s coping skills limit their ability to accept dental care. Secondly, dentists with negative personal feelings, for example, that providing care can be stressful and troublesome and that they feel time constrained.Differences in dentists’ beliefs can be partly explained by their work profile, with those treating children often, and those working under systems where they feel they can provide quality care being least likely to identify barriers to providing care for children.	Low
Pourat N, Marcus Ml,2012 (USA) [48]	3.098 generaldentists in privatepractice in California	questionnaire	Variations in dentists’ provision of services have been documented, but information about contributing factors is limited to assess variations in service provision and its correlates.	The results show variations in services provided by general dentists in private practice. Multiple factors, including the dentist’s sex, region of practice, employment of hygienists, patients’ race and population income in the area of practice were significantly and independently associated with provision of services.	High
Razak IA & Lind, 1994(Malaysia) [49]	1371 Professionally trained dentists whose names appeared in the Government Gazetle of 1990 as having been granted an Annual Practicing Certificate to practice dentistry in Malaysia in l990.	questionnaire	To examine the attitudes of Malaysian dentists toward patient education and reventive dentistry and the level of preventive care adopted in Malaysian dental practice.	Generally the Malaysian dentists had positive attitudes towards patient education and preventive dentistry including fluoridation. However, a sizable proportion of them considered that preventive measures were no challenge for the dentist. The common preventive measures given to patients were scaling, dental health education, prophylaxis and instruction in correct brushing and flossing in as much as 40 to 50 percent of the queried dentist claimed that these preventive items were provided to most or all of their new patients. In spite of the fact that the majority of the dentists had good knowledge about the application and effects of sealants only about 41% of the dentists claimed to have used sealants.	High
Riley III JLet al, 2011(USA) [50]	393 male and 73 female general dentists who were members of The Dental Practice-Based Research Network (DPBRN) and practiced within the USA	questionnaire	A number of articles have addressed differences in productivity between male and female dentists, but little is known about differences between the sexes in practice patterns regarding caries management.	Female dentists recommended at-home fluoride to a significantly larger number of their patients than did male dentists, whereas male dentists had a preference for using in-office fluoride treatments with pediatric patients. Female dentists also chose to use preventive therapy more often at earlier stages of dental caries. There were few differences between the sexes in terms of methods, time spent on or charges for restorative dentistry, and business of the practice. The practice patterns of female dentists suggest a treatment philosophy with a greater focus on caries prevention.	Low
Riley III JLet al, 2010(USA) [51]	467 general dentists in the DPBRN who practice within the United States and treat both pediatric and adult patients	questionnaire	To test the frequency of dentists’ recommendations for and use of caries-preventiveagents for children as compared with adults.	General dentists use in-office caries-preventive agents more commonly with their pediatric patients than with their adult patients. General dentists should consider providing additional in-office caries-preventive agents for their adult patients who are at increased risk of experiencing dental caries.	Low
Riley III JLet al, 2010(USA) [52]	564 practitioners inDPBRN, a multi-regionconsortium of participating practices and dental organizations.	questionnaire	To identify factors that are significantly associated with dentists’ use of specific caries preventive agentsin adult patients.	Caries prevention is commonly used with adult patients. However, the results suggest that only a subset of dentists base preventive treatments on caries risk at the individual patient level.	Low
Rock WP & Bradnockl, 1976 (Wales and England) [53]	885 dentists - Every 10th name on each list of dental practioners who had undertaken to provide general dental services in England and Wales.	mailedquestionnaire	To discover the numbers of dentists who were using preventive methods and also to gather information about the attitude of the profession towards a suggested inclusion of fees for preventive therapy on the National Health Service scale	The majority of dentists were in favor of fees for preventive treatment. It may be argued that use of preventive measures on a large scale would be an investment for the future since the need for conservative treatment would be reduced. Cost of application could be reduced by employment of dental hygienists.	Low
Serrano AGet al, 1990(Spain) [54]	1019 dental professionalsin western and easternAndalucía, and members of the Spanish Society of Preventive and communitary Odontoestomatogy	questionnaire	To understand the attitudes, knowledge and behavior of 3 groups of Spanish dentists on methods of caries prevention.	Dental professionals should elaborate and participate more in preventive and educational for the population.	High
Sesma Net al, 2006(Brazil) [55]	400 dentists ofSão Paulo city	questionnaire	To identify dentistsprofile in theprevention ofdental caries andgingival diseases	1– The vast majority (97.6%) confirm the practice of some caries and giginval disease, but only 0.3% employ the six methods analysed in this study. 2–Time since graduation influenced the practice of prevention. Those graduated in the previous 5 years employ prevention method less often. 3– Women dentits employ prevention methods more often than men dentits. 4– Dentits that received specific traning on prevention are more likely to employ it.	High
Silva RPet al, 2006(Brazil) [56]	233 Dental Surgeonswho was registeredwith CROMG (June/2002)and resident in the municipality of Lavras (Minas Gerais, Brazil)	questionnaire	To evaluate the level of Dental Surgeons’ (DSs) knowledge and clinical application of scientific evidence in Dentistry, in the city of Lavras (Minas Gerais, Brazil)	1- men, graduated from state universities were shown to be confident about prescribing and applying fluoride gel in children as a result of the diagnosed risk of dental caries; 2- those graduated up to 10 years before and attended in private dental office, were shown to be confident about prescribing and applying sealants to pit and fissure for the prevention of dental caries; 3- those that have post-graduation courses were shown to be confident to prescribe and apply chlorhexidine with a view to preventing dental caries; 4- those that graduated from state universities were shown to be more confident of monitoring white spot lesions with a view to the non-progression of dental caries; 5- men were shown to beconfident about prescribing and applying ART in children for the treatment of cavitated dental caries lesions.	Low
Tomlinson P & Treasure E, 2006(Wales) [57]	400 dentists currentlypractising in Wales	mailedquestionnaire.	To identify the attitudes of practitioners to the use of three adult preventive codes.	Few dentistsprovide preventive care to adults under the existing remuneration system. Work is necessary to enable dentists to use effective preventive techniques for adult patients.These results can be considered to show the baseline provision of prevention and could facilitate the evaluation of any changes to the current system.	Low
Tryon AM et al, 1974(USA) [58]	1020 activelypracticing dentistsin Connecticut(General practice)	questionnaire	To report on additional data on the quantity and quality of preventive services provided indental practice.	The present study only provided fragmentary evidence on the distribution of practice effort for prevention. Future studies may cast more light on some of the factors that influences dentists’ decisions to change from a curative to preventive orientation.	High
Tseveenjav B et al, 2004(Mongolia) [59]	250** All dentistspractising inUlaanbaatar,in May 2000.	questionnaire	To investigate caries-preventivemeasures (CPMs) applied by dentists in Mongolia to their own children in relation to the dentist-parents’ professional and preventive care-related backgrounds and the children’s dental health.	Caries-preventive measures applied to dentists’ children should be improved, especially in regard to sugar consumption.Comprehensive efforts are called for, stressing modern CPMs. Both the undergraduate curriculum and the continuing education program need to emphasize the use of modern methods of caries prevention.	High
Tseveenjav B et al, 2005(Mongolia) [60]	250** All dentists practising in Ulaanbaatar, in May 2000.	questionnaire	To study barriers to providing oral health education (OHE) to their patients among Mongolian dentists	Despite appreciation of OHE, Mongolian dentists seem to face practical barriers to providing oral helth education activities	Low

NA: Not available.

*Mixed methods studies: qualitative and survey

**Same population; number of participants counted only once

The included qualitative studies and surveys covered a wide geographical area with a total of 28 countries, including Australia (nine publications) [14], [15], [18], [25]–[29], [33], United States of America (nine publications) [21], [22], [31], [34], [39], [43], [44], [48], [58], England (six publications) [17], [19], [20], [30], [40], [42], Wales (four publications) [16], [23], [32], [57], Brazil (three publications) [24], [55], [56], Iran (three publications) [36] – [38], Mongolia (two publications) [59], [60] and Spain [54], Finland [45], Korea [8], Malaysia [49], Northern Ireland [35] and Romania [46] (1 publication each). Six publications [41], [47], [50]–[53] were multicenter studies or surveys involving participants from Denmark, Iceland, Norway, Sweden, Belgium, China, Czech Republic, Germany, Ireland, Madagascar, México, Singapore, South Africa, Tanzania, and Thailand.

### Quality assessment

**Figure 2:**
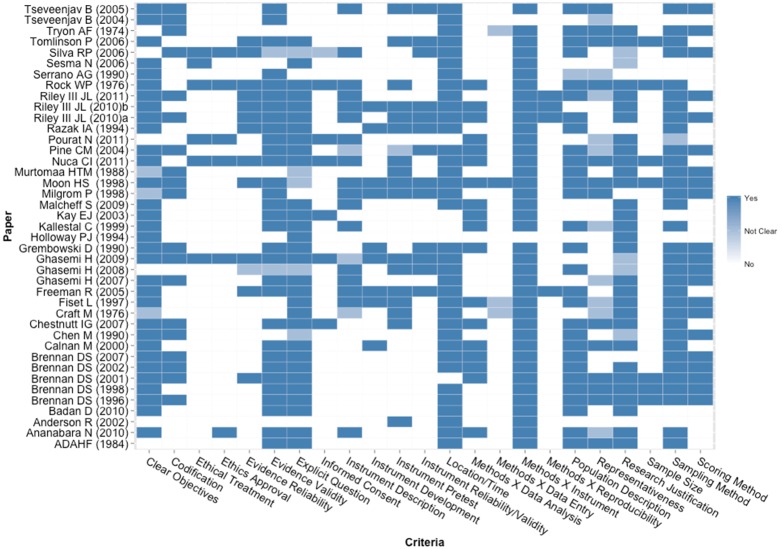
Heat map showing a gradient of quality indicators for each individual survey included in the analysis. Colors vary from white (No), light blue (Not Clear) and blue (Yes) representing the three categories used in the quality assessment.

**Figure 3:**
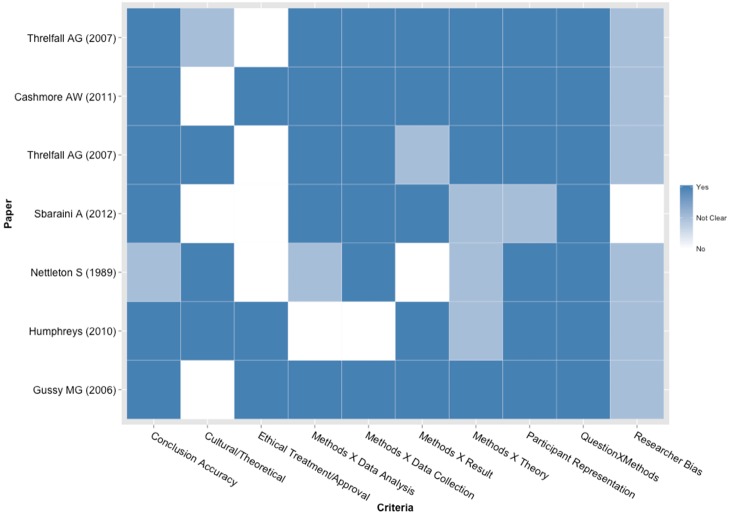
Heat map showing a gradient of quality indicators for each individual qualitative study included in the analysis. Colors vary from white (No), light blue (Not clear) and blue (Yes) representing the three categories used in the quality assessment.

Heat maps showing the gradient of quality indicators for each individual survey and qualitative study included in the analysis are shown in Figures 2 and 3, respectively. Most surveys did not present many of the quality items assessed. A total of 24 studies were judged to present high risk of bias, while 17 studies presented low risk of bias (Table 2). All the qualitative studies, on the other hand, included in the analysis presented the majority of the quality items investigated, with 6 studies deemed to have low risk of bias and only 1 considered to have moderate risk of bias (Table 1).

### Frequency Effect size

After analysis and codification of the 48 included publications, a total of 43 relevant findings were extracted. Findings were then grouped together according to categories which were judged to be topically similar. Grouped findings and their calculated frequency ES are presented in Table 3. The categories of findings to affect dentists’ motivation to perform or not preventive measures in their patients involved: dentists’ dental education and training, personal beliefs on prevention, remuneration, work conditions, gender, place of residence and also the factors that dentists believed to drive patients towards or away from performing preventive measures.

**Table 3:** Abstracting, formatting, grouping in categories, and frequency effect sizes (ES) of findings.

Driving dentiststowards performingdental cariespreventive measures	ES%	Driving dentists away from performing dental caries preventive measures	ES%
**Education and Training**			
Time since graduation(Tseveenjav, 2004; Ghasemi, 2007;Sesma, 2006; Razak, 1994;Murtomaa, 1988)	10	Time since graduation (Nettleton, 1989; Sbaraini, 2012; Tseveenjav, 2005; Riley, 2011; Riley, 2010^49^; Moon, 1988; Rock, 1974; Chen M, 1990; Riley, 2010^50^; Brennan, 1996; Brennan, 2001)	22
Communication and healtheducation skills (Threlfall, 2007^25^;Sbaraini, 2012;Tseveenjav, 2004;Milgrom, 1988; Riley, 2010^50^)	10	Lack of communication and health education skills (Nettleton, 1989; Humphreys, 2010; Murtomaa, 1988)	6
Post-graduation (Tseveenjav, 2004;Ananaba, 2010; Moon, 1988;Sesma, 2006; Chen, 1990; Kay,2003)	12	Lack of technical skill/knowledge (Fiset, 1997; Tseveenjav, 2005; Sesma, 2006; Murtomaa, 1988; Badan, 2006; Sbaraini, 2012)	12
Graduation from public dentalschools (Silva, 2006; Badan, 2006)	4	Professional specialization (Humphreys, 2010; Sesma, 2006; Murtomaa, 1988)	6
Ongoing Education/Traning(Sbaraini, 2012;Ghasemi, 2007;Chen, 1990; Ghasemi, 2008; Kay,2003)	10	Biologicism (Threlfall, 2007^16^; Nettleton, 1989; Humphreys, 2010; Sbaraini, 2012; Craft, 1976; Serrano, 1990; Tseveenjav, 2005; Ananaba, 2010; Malcheff, 2009; Sesma, 2006; Murtomaa, 1988; Calnan, 2000; Badan, 2006)	27
Participation in discussiongroups/networks (Threlfall, 2007^17^;Sbaraini, 2012; Tryon, 1974;Tseveenjav, 2004)	4	Lack of educational material (Threlfall, 2007^16^; Tseveenjav, 2005; Rock, 1974; Murtomaa, 1988; Badan, 2006)	10
Complementary reading (Silva,2006; Ghasemi, 2008)	4	Difficulty working with children (Pine, 2004)	2
**Personal Beliefs**			
Personal satisfaction (Holloway,1994; Craft, 1976; Calnan, 2000)	6	Disbelief in fluoride effect (Threlfall, 2007^17^; Craft, 1976)	4
Professional understanding of thebenefits (Holloway, 1994;Threlfall, 2007^16^; Threlfall, 2007^17^;Nettleton, 1989; Sbaraini, 2012)	10	Lack of professional understanding of the benefits (Sbaraini, 2012; ADAHF, 1984; Tomlinson, 2006; Murtomaa, 1988; Holloway, 1994; Calnan, 2000)	12
Positive cost/benefit ratio(Holloway, 1994; Sbaraini, 2012;Murtomaa, 1988)	6	Negative cost/benefit ratio (Ananaba, 2010; Malcheff, 2009; Razak, 19944; Murtomaa, 1988)	8
		Lack of interest in the activity (Nettleton, 1989)	2
		Depreciation of the professional image (Nettleton, 1989; Ghasemi, 2007; Murtomaa, 1988)	6
**Work conditions**			
Work in the public health system(Tseveenjav, 2004;Moon, 1988; Anderson, 2002;Chestnut, 2007)	8	Work in the public health system (Tseveenjav, 2005; Freeman, 2005; Riley, 2011; Badan, 2006)	8
Team work (Holloway, 1994; Threlfall, 2007^16^;Tryon, 1974; Craft, 1976; Chen,1990; Murtomaa,1988; Grembowsky, 1990;Freeman, 2005;Cashmore 2011)	21	Work should be performed by dental technicians/assistants (Threlfall, 2007^18^; Nettleton, 1989; Anderson, 2002; Murtomaa, 1988)	8
Presence of dental caries(Threlfall, 2007^16^; Humphreys, 2010)	4	Difficulty working with children (Pine, 2004)	2
**Remuneration**			
Coverage by private health insurance(Kay, 2003; Brennan, 2003; Riley, 2010^49^)	6	Lack of coverage by private health insurance (Ghasemi, 2009; Fiset, 1997; Tomlinson, 2006; Calnan, 2000)	8
		Low pay (Threlfall, 2007^18^; Sbaraini, 2012; Fiset, 1997; Craft, 1976; Serrano, 1990; Pine, 2004; Milgrom, 1988; Ghasemi, 2007; Razak, 1994; Murtomaa, 1988; Calnan, 2000; Grembowsky, 1990)	25
**Gender**			
Male dentists (Riley, 2011; Moon, 1988;Razak, 1994;Ghasemi, 2009; Silva, 2006)	10	Male dentists (Nettleton, 1989; Ghasemi, 2007; Sesma, 2006; Ghasemi, 2008; Riley, 2010^49^; Riley, 2010^50^; Brennan, 2001; Pourat, 2012; Riley, 2011^51^)	19
**Residence**			
Living in the rural area(Moon, 1988)	2	Living in the rural area (Ghasemi, 2007; Ghasemi, 2009; Rock, 1974; Chen, 1990; Brennan, 2001; Brennan, 2003; Brennan, 2007)	15
**Patients**			
Parents’ motivation(Gussy, 2006; Threlfall, 2007^16^;Threlfall, 2007^17^)	4	Lack of awareness (Gussy, 2006; Nettleton, 1989; Ananaba, 2010; Malcheff, 2009; Ghasemi, 2009; ADAHF, 1984; Murtomaa, 988; Badan, 2006)	17
Patients’ age(Tomlinson, 2006; Threlfall, 2007^16^)	4	Lack of motivation (Gussy, 2006; Threlfall, 2007^16^; Nettleton, 1989; Humphreys, 2010; Murtomaa, 1988)	8
		Fear (Gussy, 2006; Pine, 2004; Calnan, 2000)	6
		Cost (Gussy, 2006; Murtomaa, 1988; Kay, 2003)	6
		Age – small children (Humphreys, 2010; Pine, 2004; Milgrom, 1988; Ananaba, 2010; Malcheff, 2009; Brennan, 1996)	12
		Embarrassment (Nettleton, 1989; Tseveenjav 2005; Murtomaa 1988)	6

To conserve space, only the first author is listed.

**Figure 4:**
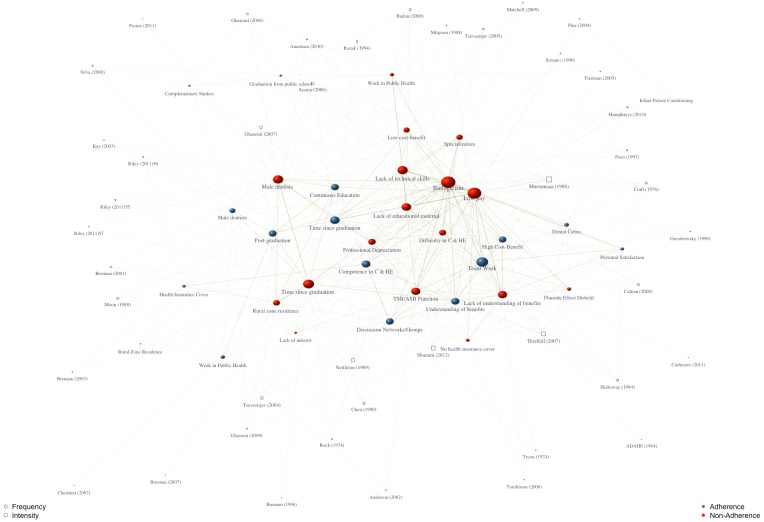
Factors that drive dentists towards or away from performing preventive measures. Graph theory-based figure showing the relation among qualitative studies and surveys included in the metasummary model. Squares represent the individual studies included, and circles the emerging factors. Size of each individual marking indicates its effect size (ES) in the model; larger markings being more recurrent. Studies presenting lower intensity ES (prevalence) appear further from the center, while studies with higher intensity ES closer to the center of the figure.

The findings with the highest frequency ES to drive dentists away from performing preventive measures were “biologicism”, (27%), “low pay” (25%), “time since graduation” (22%), and “male dentists” (19%). Whereas, “team work” (21%), “post-graduation” (12%), and “professional understanding of the benefits” (12%) were identified as the main reasons for dentists adherence to preventive measures. The factors for dentists’ adherence or non-adherence to dental caries preventive measures are graphically shown in Figure 4.

**Figure 5:**
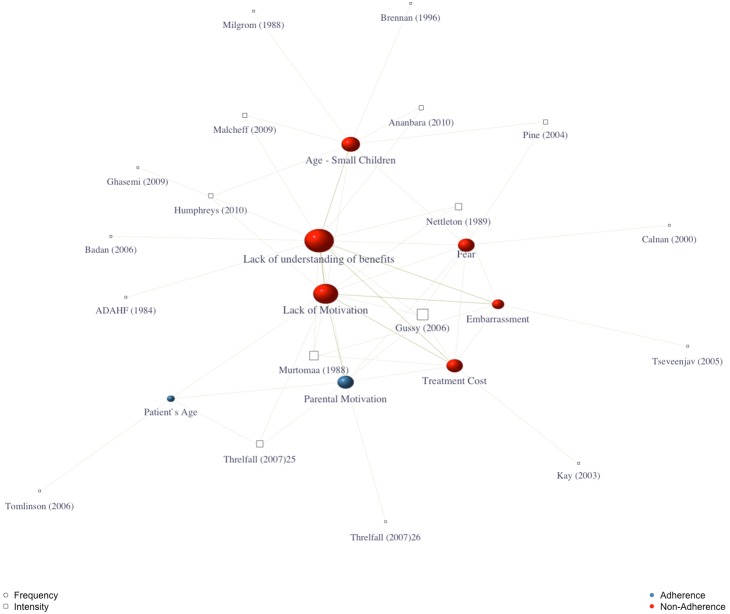
Factors dentists believed to drive patients towards or away from performing preventive measures. Graph theory-based figure showing the relation among surveys included in the metasummary model. Squares represent the individual studies included, and circles the emerging factors. Size of each individual marking indicates its effect size in the model; larger markings being more recurrent. Studies presenting lower intensity ES (prevalence) appear further from the center, while studies with higher intensity ES closer to the center of the figure.

The main factor that dentists believed keep patients from performing preventive measures were “lack of understanding of the benefits” (17%), “age/small children” (12%), and “patient lack of motivation” (8%). While “parents’ motivation” and “patients’ age” (4%) were the reasons to lead to patients towards the same preventive measures. Figure 5 graphically illustrates the factors that dentists believed to drive patients towards or away from performing preventive measures.

### Intensity effect sizes

Calculated intensity ES are presented in Table 4. The publication that presented the highest intensity ES, i.e., that presented the highest number of themes relative to the total number of themes, was Murtomaa [43] with a score of 40%, followed by Nettleton [14] and Sbaraini [15] with 26%, and Threlfall [16], [17] with 23%. Among the 48 selected publications, 18 had scores between 8% and 22%, and 26 publications had scores below 8%. Only one finding (biologicism) presented effect size >25%, which resulted in intensity ES >25% = 100% in 13 studies, while intensity ES for the remaining studies was 0%.

**Table 4:** Intensity effect sizes (ES) in relation to all themes and themes with frequency effects sizes >25%.

Report	IntensityES(%)	IntensityES>25% (%)	Report	IntensityES (%)	IntensityES>25% (%)
ADAHF (1984)^18^	7	*	Kay EJ (2003)^39^	9	*
Ananaba N (2010)^19^	9	100	Malcheff S (2009)^40^	12	100
Anderson R (2002)^20^	5	*	Milgrom P (1988)^41^	7	*
Badan DE (2006)^21^	14	100	Moon HS (1988)^42^	12	*
Brennan DS (1996)^22^	2	*	Murtomaa HTM (1988)^43^	40	100
Brennan DS (1998)^23^	2	*	Nettleton S (1989)^14^	26	100
Brennan DS (2001)^24^	7	*	Nuca CI (2011)^44^	2	*
Brennan DS (2003)^25^	5	*	Pine CM (2004)^45^	9	*
Brennan DS (2007)^26^	2	*	Pourat N (2011)^46^	2	*
Calnan M (2000)^27^	12	100	Razak I (1994)^47^	12	*
Cashmore AW (2011)^11^	2	*	Riley III RL (2011)^48^	7	*
Chen M (1990)^28^	12	*	Riley III RL (2010)^49^	7	*
Chestnut IG (2007)^29^	2	*	Riley III RL (2010)^50^	3	*
Craft M (1976)^30^	14	100	Rock WP (1976)^51^	7	*
Fiset L (1997)^31^	7	*	Sbaraini A (2012)^15^	26	100
Freeman R (2005)^32^	2	*	Serrano AG (1990)^52^	5	100
Ghasemi H (2007)^33^	14	*	Sesma N (2006)^53^	14	100
Ghasemi H (2008)^34^	7	*	Silva RP (2006)^54^	7	*
Ghasemi H (2009)^35^	9	*	Threlfall AG (2007)^16^	23	100
Grembowsky D (1990)^36^	5	*	Threlfall AG (2007)^17^	7	*
Gussy MG (2006)^12^	9	*	Tomlinson P (2006)^55^	7	*
Holloway PJ (1994)^37^	12	*	Tryon F (1974)^56^	2	*
Humphreys RE (2010)^13^	14	100	Tseveenjav B (2004)^57^	7	*
Kallestål C (1999)^38^	5	*	Tseveenjav B (2005)^58^	14	100

*Reports that did not obtain themes with frequency effects sizes >25%.

## Discussion

This systematic review and metasummary of qualitative studies and surveys analyzed factors that drive dentists towards or away from dental caries preventive measures.

Surveys and qualitative research differ in how data are obtained. The minimally structured and open-ended interviewing style typically associated with qualitative studies allows an unlimited number of responses, yielding data with a wider range of responses concerning a target event. In contrast, the highly structured and closed-ended questionnaire typically associated with surveys limits the number and specifies the nature and direction of responses, producing data with a narrower range of responses. Seeing all of the findings belonging to one topic together preserves the complexity of the findings. The methodology used in this systematic review allowed the aggregation and interpretation of descriptive findings, which were comparable among themselves [13]. A diversity of findings were abstracted from the selected studies (Table 3) that were analyzed and their potential relevance in understanding which factors drive dentists towards or away from performing preventive measures were commented.

The findings of this systematic review indicate that the reasons for dentists’ adherence to providing prevention are multifactorial and dependent on how and where the study was performed. Nonetheless, it is important to point out the limitations imposed by the quality of the selected reports. The lack of standardization, together with a lack of adequate description of the study methodology, negatively affected the quality assessment, with most of the selected surveys (58%) included in this review being judged to have a high risk of bias. This clearly demonstrate that studies following well established criteria for the conduction of surveys with validated instruments are necessary to better understand dentists motivation or lack of motivation towards preventive measures.

Qualitative studies, on the other hand, were judged to present low risk of bias, with most studies presenting the items analyzed. The drawback concerning this type of study was that fact only a handful of reports were retrieved from the literature. The advantage of qualitative studies is that, due to its design, they may bring to the surface perceptions, feelings, and opinions that are sometimes impossible to be captured by surveys. The included qualitative studies covered just three countries (England, Wales and Australia), limiting the generalizability of findings. Well-designed qualitative studies performed in lower-income countries would significantly add to the understanding of this matter.

A high percentage of studies (54%) had a low intensity frequency, indicating low prevalence of the findings (Table 4). This limitation was compensated by the diversity of findings found in the studies. This multiplicity of findings accounting for dentists’ attitude towards prevention abstracted from the selected reports may be explained by the methodological variability of the reports and the wide geographic area covered.

Nonetheless, despite the low calculated frequency (Table 3) and intensity (Table 4) effect sizes, two main categories of findings have emerged as being relevant to the reasons for adherence or non-adherence to preventive measures. Dental education and training has emerged as the most important category to affect dentists’ attitude to their perception of how to conduct their activities. It seems clear that when dentists are continuously engaged in their professional and educational development, the more open they are to the new demands of the profession, and more likely to embrace prevention in their daily routine [31], [36], [55], [59], [60]. As a result, their education and training have a direct effect on their personal beliefs and vision of prevention as something beneficial for the patient with associated professional satisfaction. In contrast, however, the ways dentists are being remunerated for dental caries preventive measures need to be examined more carefully. The findings in this study demonstrated that low or no remuneration for preventive measures may be an important hindrance to their motivation. This is in agreement with the findings of a recent Cochrane revision, which have indicated that financial incentives within remuneration systems may produce changes to clinical activity undertaken by primary care dentists [61]. Thus, a combination of continuous education and training coupled to an acceptable pay scheme would seem to be a reasonable approach to increase dental professionals’ adherence to dental caries preventive measures.

It is expected that this study may contribute to the understanding of factors that can drive dentists towards or away from performing dental caries preventive measures. Moreover, this information may then be used as a useful reference for planning and decision making aimed at changing dental practice and improving the oral health care provided to the general population.

## Supporting Information

Appendix S1 **Search strategy used in PubMed.**
(DOCX)

Checklist S1 **PRISMA Checklist.**
(DOC)

## References

[1] World Health Organization (2012) Oral Health Fact Sheet. n. 318, April 2012. Available: http://www.who.int/mediacentre/factsheets/fs318/en/. Accessed: 2013 Mar 01.

[2] Beltrán-Aguilar ED, Barker LK, Canto MT, Dye BA, Gooch BF, et al (2005) Surveillance for dental caries, dental sealants, tooth retention, edentulism, and enamel fluorosis–United States, 1988–1994 and 1999–2002. MMWR Surveill Summ 54: 1–43. 16121123

[3] World Health Organization (2014) What is the burden of oral disease? Available: http://www.who.int/oral_health/disease_burden/global/en/. Accessed: 2014 Aug 16.

[4] Petersen PE, Bourgeois D, Ogawa H, Estupinan-Day S, Ndiaye C (2005) The global burden of oral diseases and risks to oral health. Bull World Health Organ [online] 83: 661–669. PMC2626328 16211157

[5] Yee R, Sheiham A (2002) The burden of restorative dental treatment for children in Third World countries. Int Dent J 52: 1–9. 11931216

[6] Petersen PE (2003) The World Oral Health Report 2003: continuous improvement of oral health in the 21st century – the approach of the WHO Global Oral Health Programme. Community Dent Oral Epidemiol 31: 3–24. 15015736 10.1046/j..2003.com122.x

[7] Nadanovsky P, Sheiham A (1995) Relative contribution of dental services to the changes in caries levels of 12-year-old children in 18 industrialized countries in the 1970s and early 1980s. Community Dent Oral Epidemiol 23: 331–339. 8681514 10.1111/j.1600-0528.1995.tb00258.x

[8] Moon HS, Paik DI, Horowitz AM, Kim JB (1998) National survey of Korean dentists’ knowledge and opinions: Dental caries etiology and prevention. J Public Health Dent 58: 51–56. 9608446 10.1111/j.1752-7325.1998.tb02990.x

[9] Moher D, Liberati A, Tetzlaff J, Altman DG (2009) The PRISMA Group (2009) Preferred reporting items for systematic reviews and meta-analyses: the PRISMA statement. PLoS Med 6(7): e1000097 10.1371/journal.pmed.1000097 19621072 PMC2707599

[10] Shea BJ, Grimshaw JM, Wells GA, Boers M, Andersson N, et al (2009) Development of AMSTAR: A measurement tool to assess the methodological quality of systematic reviews. BMC Med Res Methodol 7: 10. 10.1186/1471-2288-7-10 PMC1810543 17302989

[11] Bennett C, Khangura S, Brehaut JC, Graham ID, Moher D, et al (2011) Reporting Guidelines for Survey Research: An Analysis of Published Guidance and Reporting Practices. PLoS Med 8(8): e1001069 10.1371/journal.pmed.1001069 PMC3149080 21829330

[12] The Joanna Brings Institute. Available: http://joannabriggs.org/sumari.html. Accessed: 2014 Sep 16.

[13] Sandelowski M, Barroso J, Voils CI (2007) Using qualitative metasummary to synthesize qualitative and quantitative descriptive findings. Res Nurs Health 30: 99–111. 17243111 10.1002/nur.20176 PMC2329806

[14] Cashmore AW, Noller J, Ritchie J, Johnson B, Blinkhorn AS (2011) Reorienting a paediatric oral health service towards prevention: Lessons from a qualitative study of dental professionals. Health Promot J Austr 22: 17–21. 21717832 10.1071/he11017

[15] Gussy MG, Waters E, Kilpatrick NM (2006) A qualitative study exploring barriers to a model of shared care for pre-school children’s oral health. Br Dent J 201: 165–170. 16902551 10.1038/sj.bdj.4813849

[16] Humphreys RE, Richards W, Gill P (2010) Perceptions of first year foundation dentists on oral health education and its role in general dental practice. Br Dent J 209: 601–606. 21169964 10.1038/sj.bdj.2010.1133

[17] Nettleton S (1989) Dentists and dental health education: a study of the perceptions of 28 community dentists. Community Dent Health 6: 47–60. 2655844

[18] Sbaraini A (2012) What factors influence the provision of preventive care by general dental practitioners? Br Dent J 212: 1–9. 22677875 10.1038/sj.bdj.2012.498

[19] Threlfall AG, Hunt CM, Milsom KM, Tickle M, Blinkhorn AS (2007) Exploring factors that influence general dental practitioners when providing advice to help prevent caries in children. Br Dent J 202: 1–4. 17308533 10.1038/bdj.2007.143

[20] Threlfall AG, Milsom KM, Hunt CM, Tickle M, Blinkhorn AS (2007) Exploring the content of the advice provided by general dental practitioners to help prevent caries in young children. Br Dent J 202: 1–4. 17256013 10.1038/bdj.2007.46

[21] American Dental Association Health Foundation (1984) Prevention in the dental office: results of a preventive dentistry survey. J Am Dent Assoc 108: 809, 811–812. 10.14219/jada.archive.1984.0085 6588124

[22] Ananaba N, Malcheff S, Briskie D, Inglehart MR (2010) Infant oral health examinations: attitudes and professional behavior of general and pediatric dentists in Michigan and pediatric dentists in the U.S. J Mich Dent Assoc. 92: 38–43. 21291093

[23] Anderson R, Treasure ESAS (2002) Oral health promotion practice: a survey of dental professional in Wales. Int J Health Promot Educ 40: 9–14.

[24] Badan DE, Marcelo VC, Rocha DG (2010) Perceptions and use of collective health by surgeon dentists egress from Federal University of Goiás. Cien Saude Colet 15: 1811–1818. 20640343 10.1590/s1413-81232010000700093

[25] Brennan DS, Spencer AJ, Szuster FS (1996) Dentist service rates and distribution of practice styles over time. Community Dent Oral Epidemiol 24: 145–51. 8654037 10.1111/j.1600-0528.1996.tb00832.x

[26] Brennan DS, Spencer AJ, Szuster FS (1998) Service provision trends between 1983–84 and 1993–94 in Australian private general practice. Aust Dent J 43: 331–336. 9848985 10.1111/j.1834-7819.1998.tb00184.x

[27] Brennan DS, Spencer AJ (2001) Practice belief scales among private general dental practitioners. Aust Dent J 46: 186–193. 11695157 10.1111/j.1834-7819.2001.tb00280.x

[28] Brennan DS, Spencer AJ (2003) Provision of diagnostic and preventive services in general dental practice. Community Dent Health 20: 5–10. 12688597

[29] Brennan DS, Spencer AJ (2007) Trends in private dental service provision in major city and other Australian locations. Aust J Rural Health 15: 189–95. 17542792 10.1111/j.1440-1584.2007.00883.x

[30] Calnan M, Silvester S, Manley G, Taylor-Gooby P (2000) Doing business in the NHS: exploring dentists’ decisions to practise in the public and private sectors. Sociol Health Illn 22: 742–764.

[31] Chen M (1990) Preventive dentistry in Texas, USA. Community Dent Oral Epidemiol 18: 239–43. 2147406 10.1111/j.1600-0528.1990.tb00067.x

[32] Chestnut IG, Thomas DR, Patel R, Treasure ET (2007) Perceptions and attitudes to a fundamental reform of general dental services in Wales. Prim Dent Care 14: 13–18. 17288719 10.1308/135576107779398228

[33] Craft M, Sheiham A (1976) Attitudes to prevention amongst dental practitioners. A comparison between the North and South of England. Br Dent J 141: 371–6. 1070321 10.1038/sj.bdj.4803850

[34] Fiset L, Grembowski D (1997) Adoption of innovative caries-control services in dental practice: a survey of Washington state dentists. J Am Dent Assoc 128: 337–345. 9066218 10.14219/jada.archive.1997.0197

[35] Freeman R, Kerr G, Salmon K, Speedy P (2005) Patient-active prevention in primary dental care: a characterization of general practices in Northern Ireland. Prim Dent Care 12: 42–46. 15901431 10.1308/1355761053695185

[36] Ghasemi H, Murtomaa H, Torabzadeh H, Vehkalahti MM (2007) Knowledge of and attitudes towards preventive dental care among Iranian dentists. Eur J Dent 1: 222–229. 19212471 PMC2609918

[37] Ghasemi H, Murtomaa H, Torabzadeh H, Vehkalahti MM (2008) Risk-based approach in preventive practice among Iranian dentists. Oral Health Prev Dent 6: 53–60. 18399308

[38] Ghasemi H, Murtomaa H, Torabzadeh H, Vehkalahti MM (2009) Perceived barriers to the provision of preventive care among Iranian dentists. Oral Health Prev Dent 7: 339–346. 20011751

[39] Grembowski D, Milgrom P, Fiset L (1990) Factors influencing variation in dentist service rates. J Public Health Dent 50: 244–250. 2118182 10.1111/j.1752-7325.1990.tb02130.x

[40] Holloway PJ, Clarkson JE (1994) Cost: benefit of prevention in practice. Int Dent J 44: 317–322. 7822057

[41] Källestål C, Wang NJ, Petersen PE, Arnadottir IB (1999) Caries-preventive methods used for children and adolescents in Denmark, Iceland, Norway and Sweden. Community Dent Oral Epidemiol 27: 144–151. 10226725 10.1111/j.1600-0528.1999.tb02004.x

[42] Kay EJ, Ward N, Locker D (2003) A general dental practice research network: impact of oral health in general dental practice patients. Br Dent J 194: 621–625. 12819698 10.1038/sj.bdj.4810259

[43] Malcheff S, Pink TC, Sohn W, Inglehart MR, Briskie D (2009) Infant oral health examinations: pediatric dentists’ professional behavior and attitudes. Pediatr Dent 31: 202–209. 19552224

[44] Milgrom P, Weinstein P, Chapko M, Grembowski D, Spadafora A (1988) Dentists’ attitudes and behaviors in counseling patients about oral self-care. J Am Coll Dent 55: 48–53. 3164012

[45] Murtomaa H, Telivuo M (1988) The attitudes of Finnish dentists in private practice towards dental health education. Community Dent Health 5: 369–380. 3214793

[46] Nuca CI, Amariei CI, Paris SD (2011) Preventive Dentistry: current working practices of dentists from the south-eastern region of Romania. OHDM 10: 131–142.

[47] Pine CM, Adair PM, Burnside G, Nicoll AD, Gillett A, et al (2004) Barriers to the treatment of childhood caries perceived by dentists working in different countries. Community Dent Health 21: 112–120. 15072480

[48] Pourat N, Marcus M (2011) Variations in self-reported provision of services by general dentists in private practice. J Am Dent Assoc 142: 1050–1060. 21881073 10.14219/jada.archive.2011.0327

[49] Razak I, Lind OP (1994) Patient education and preventive care in Malaysian dental practice. J Clin Pediatr Dent 18: 313–322. 7811664

[50] Riley JL 3rd, Gordan VV, Rouisse KM, McClelland J, Gilbert GH (2011) Differences in male and female dentists’ practice patterns regarding diagnosis and treatment of dental caries: findings from The Dental Practice-Based Research Network. J Am Dent Assoc 142: 429–440. 21454850 10.14219/jada.archive.2011.0199 PMC3079556

[51] Riley JL 3rd, Richman JS, Rindal DB, Fellows JL, Qvist V, et al (2010) Use of caries-preventive agents in children: findings from the Dental Parctice-Based Research Network. Oral Health Prev Dent 8: 351–359. 21180672 PMC3074637

[52] Riley JL 3rd, Gordan VV, Rindal DB, Fellows JL, Ajmo CT, et al (2010) Preferences for caries prevention agents in adult patients: findings from the dental practice-based research network. Community Dent Oral Epidemiol 38: 360–370. 20560997 10.1111/j.1600-0528.2010.00547.x PMC2933181

[53] Rock WP, Bradnock G (1976) Preventive dentistry in general practice. A study of current use. Br Dent J 140: 61–64. 1061600 10.1038/sj.bdj.4803703

[54] Gonzales Serrano A, Cenizo Alcaide JA, Lopez Bermejo MA (1990) Attitudes, knowledge and behavior of 3 groups of Spanish dentists on methods of caries prevention. Rev Eur Odontoestomatol 2: 107–110. 2222659

[55] Sesma N, Alves AP, Telles FBA, Macedo MO, Santos PM, et al (2006) O perfil dos cirurgiões-dentistas da cidade de São Paulo na prática da prevenção de cáries e doenças gengivais. Revista de Odontologia da Universidade Cidade de São Paulo 18: 257–263.

[56] Silva RP, Loureiro CAS, Pereira CV, Flório FM (2006) Profile of the dental surgeon related to the recommendation of individual preventive strategies. Braz J Oral Sci 5: 1022–1027.

[57] Tomlinson P, Treasure E (2006) Provision of prevention to adults in NHS dental practices and attitudes to prevention. Br Dent J 200: 393–397. 16607332 10.1038/sj.bdj.4813425

[58] Tryon AF (1974) An analysis of preventive dental activities in general practice. J Am Soc Prev Dent 4: 20–25. 4529870

[59] Tseveenjav B, Vehkalahti MM, Murtomaa H (2004) Caries-preventive measures applied by Mongolian dentists to their own children. Oral Health Prev Dent 2: 203–209. 15641623

[60] Tseveenjav B, Vehkalahti MM, Murtomaa H (2005) Barriers to the provision of oral health education among Mongolian dentists. Oral Health Prev Dent 3: 183–188. 16355652

[61] Brocklehurst P, Price J, Glenny AM, Tickle M, Birch S, et al (2013) The effect of different methods of remuneration on the behaviour of primary care dentists. Cochrane Database Syst Rev 11: CD009853 10.1002/14651858. CD009853.pub2 PMC6544809 24194456

