# Supplementary material for: Factors That Drive Dentists towards or Away from Dental Caries Preventive Measures: Systematic Review and Metasummary
Source: PLoS One. 2014 Oct 8;9(10):e107831. doi: 10.1371/journal.pone.0107831 (PMC4189795; doi:10.1371/journal.pone.0107831)
Supplement: Appendix S1 — Search strategy used in PubMed. (DOCX) [file pone.0107831.s001.docx]

**Appendix S1:** Search strategy used in PubMed.

|  | Group | Key words | Search Strategy  (Mesh and entry terms) |
| --- | --- | --- | --- |
| P = Patient |  | dentist, dentists / general dental practitioner / general dental practitioners | "Dentist"[Mesh] OR "Dentists"[All Fields] OR “General Dental Practitioner” [All Fields] OR “General Dental Practitioners” [All Fields] |
| I = Intervention |  | dental caries / prevention /oral health | "Dental Caries"[Mesh] OR “Decay, Dental”[All Fields] OR “Dental Decay”[All Fields] OR “Caries, Dental”[All Fields] OR “Dental White Spot”[All Fields] OR “White Spots, Dental”[All Fields] OR “White Spots”[All Fields] OR “Spot, White”[All Fields] OR “Spots, White”[All Fields] OR “White Spot”[All Fields] OR “Dental White Spots”[All Fields] OR “White Spot, Dental”[All Fields] OR "Prevention and Control"[Subheading] OR "Preventive Therapy "[All Fields] OR "Prophylaxis"[All Fields] OR "Preventive Measures"[All Fields] OR "Prevention"[All Fields] OR "Control"[All Fields] OR "Primary Prevention"[Mesh] OR "Prevention, Primary"[All Fields] OR "Oral Health"[Mesh] |
